# Muscle Transcriptome Analysis of Mink at Different Growth Stages Using RNA-Seq

**DOI:** 10.3390/biology13050283

**Published:** 2024-04-23

**Authors:** Min Rong, Xiumei Xing, Ranran Zhang

**Affiliations:** 1 Institute of Special Animal and Plant Sciences, Chinese Academy of Agricultural Sciences, Changchun 130112, China; rongmin12@126.com (M.R.); xingxiumei2004@126.com (X.X.); 2 Dezhou Animal Husbandry and Veterinary Development Center, Dezhou 253000, China; 3 Key Laboratory of Genetics, Breeding and Reproduction of Special Economic Animals, Ministry of Agriculture and Rural Affairs, Changchun 130112, China

**Keywords:** mink, transcriptomics, muscle development

## Abstract

**Simple Summary:**

The mink is a small and valuable fur animal resource. RNA-seq was utilized to identify key genes associated with the growth and development of mink. Consequently, genes related to embryonic development (*PEG10*, *IGF2*, *NRK*), cell cycle regulation (*CDK6*, *CDC6*, *CDC27*, *CCNA2*), and the FGF family (*FGF2*, *FGF6*, *FGFR2*) exhibited upregulation at 45 days of age in mink. This suggests their potential involvement in early growth and developmental processes. Conversely, genes associated with skeletal muscle development (*PRVA*, *TNNI1*, *TNNI2*, *MYL3*, *MUSTN1*), a negative regulator of the cell cycle gene (*CDKN2C*), and *IGFBP6* were found to be upregulated at 90 days of age in mink, indicating their potential involvement in rapid growth. In summary, our experimental data establish a foundation for the individual selection of larger-sized mink and elucidate the regulatory mechanisms underlying their growth and development.

**Abstract:**

Mink is a kind of small and precious fur animal resource. In this study, we employed transcriptomics technology to analyze the gene expression profile of mink pectoral muscle tissue, thereby elucidating the regulatory mechanisms underlying mink growth and development. Consequently, a total of 25,954 gene expression profiles were acquired throughout the growth and development stages of mink at 45, 90, and 120 days. Among these profiles, 2607 genes exhibited significant differential expression (|log2(fold change)| ≥ 2 and *p*_adj < 0.05). GO and KEGG enrichment analyses revealed that the differentially expressed genes were primarily associated with the mitotic cell cycle process, response to growth factors, muscle organ development, and insulin resistance. Furthermore, GSEA enrichment analysis demonstrated a significant enrichment of differentially expressed genes in the p53 signaling pathway at 45 days of age. Subsequent analysis revealed that genes associated with embryonic development (e.g., *PEG10*, *IGF2*, *NRK*), cell cycle regulation (e.g., *CDK6*, *CDC6*, *CDC27*, *CCNA2*), and the FGF family (e.g., *FGF2*, *FGF6*, *FGFR2*) were all found to be upregulated at 45 days of age in mink, which suggested a potential role for these genes in governing early growth and developmental processes. Conversely, genes associated with skeletal muscle development (*PRVA*, *TNNI1*, *TNNI2*, *MYL3*, *MUSTN1*), a negative regulator of the cell cycle gene (*CDKN2C*), and *IGFBP6* were found to be up-regulated at 90 days of age, suggesting their potential involvement in the rapid growth of mink. In summary, our experimental data provide robust support for elucidating the regulatory mechanisms underlying the growth and development of mink.

## 1. Introduction

The mink (*Neovison vison*) is a small fur-bearing animal renowned as the “King of Fur” due to its high-quality fur, exquisite coloration, and soft yet durable skin. After the introduction of mink in 1956, China has emerged as a leading country for mink breeding, achieving a fur harvest of 5.79 million pieces in 2022. However, in the absence of a comprehensive genetic breeding program, the responsibility of selection has been entrusted to experienced breeders. The main economic value of mink is their fur, and size is an important indicator of the grade of the fur. Under the same conditions as other indicators, the larger the fur, the higher the price, which directly affects the efficiency of the breeding farm. Furthermore, mink skin size had a strong positive genetic correlation with body length and body weight [1,2], suggesting that body weight and length measured on live animals were reliable indicators of dried fur size. Thus, studying the genetic regulatory mechanisms of mink growth is crucial for the mink breeding industry. In recent years, with the rapid development of sequencing technology, genomics has been widely used in the study of molecular regulation mechanisms of animal growth and development, promoting the process of animal genome selection breeding [3,4,5,6,7]. However, the relevant research on mink is still lagging behind. It was not until 2017 that the first draft of genome assembly for American mink was published, with a size of 2.4 GB [8]. The breeding of mink genomes becomes possible with this advancement. However, due to the lack of chromosome information, the amount of gene annotation, and other shortcomings, the study of the genome level of mink is limited. With the deepening of research and the development of technology, a chromosome-level genome of mink was assembled in 2022, with a size of 2.68 GB [9], which provides a high-quality reference genome for the study of mink growth and development mechanisms and will greatly promote the pace of mink genome selection breeding research. 

Transcriptomics plays a pivotal role in functional genomics, facilitating a comprehensive understanding of gene regulation at the transcriptional level, and serving as an efficacious approach to investigate intricate biological phenomena. Currently, numerous scholars have employed transcriptome technology to investigate the growth performance of animals. Shang performed a comparative transcriptome analysis on pigs exhibiting different phenotypes and discovered that 20 genes involved in myoblast differentiation and muscle fiber formation potentially contribute to the postnatal growth rate and body weight of pigs [10]. Wang identified seven growth-related genes through the comparative transcriptome analysis of Muscovy duck ileum tissue, providing a theoretical foundation for investigating the impact of the ileum on duck growth and metabolism [11]. Tang identified 290 and 87 differentially expressed genes associated with growth traits in the comparative transcriptome analysis of pituitary and muscle tissues from large and small geese, respectively [12,13]. Wen performed a comparative transcriptome analysis on muscle tissue from Tibetan sheep at four different growth stages, revealing that the *LIPE*, *LEP*, *ADIPOQ*, *SCD*, and *FASN* genes may modulate muscle fiber type transformation through the AMPK signaling pathway, consequently impacting meat quality [14]. Identifying these key genes related to economic traits will help uncover the molecular mechanisms governing growth and development, facilitating genome-based selective breeding strategies for animals.

In the preliminary study, we generated growth curves for mink, encompassing weight and body length as phenotypic traits. The results showed that the growth rates of weight and body length were relatively fast during the period from 45 to 120 days of age, especially from 45 to 90 days of age [15]. Therefore, in this study, the muscle tissues of silver-blue mink at three different growth stages (45 days, 90 days, and 120 days of age) were used as samples. Transcriptome sequencing technology was employed to identify key genes and signaling pathways related to muscle growth and development in mink, aiming to establish a foundation for the individual selection of larger-sized mink and to elucidate the regulatory mechanisms underlying their growth and development.

## 2. Materials and Methods

### 2.1. Samples Selection and Preparation

The silver-blue mink utilized in this study were all provided by Dalian Mingwei Marten Industry Co., Ltd. (Dalian, China). A total of nine male silver-blue mink in three litters with good body condition and a consistent feeding environment were selected as experimental animals. Each mink was individually housed in a spacious, ventilated cage within a semi-open facility, ensuring optimal comfort and minimal stress. At the ages of 45 days, 90 days, and 120 days, respectively, three male silver-blue mink were chosen from each litter. Euthanasia was performed using carbon monoxide gas following approved protocols to ensure swift and humane death. Immediately after euthanasia, breast muscle was taken using sterile scalpels, cut into small pieces, placed into frozen tubes for rapid freezing with liquid nitrogen, and brought back to the laboratory for storage at −80 °C. The experiment was conducted in accordance with the ARRIVE guidelines, and all animal experimental protocols were approved and authorized by the Animal Care and Use Committee of the Institute of Special Animal and Plant Sciences, Chinese Academy of Agricultural Sciences (permit no. ISAPSAEC-2023-032).

### 2.2. RNA Extraction and Quality Control

Total RNA was extracted from the nine samples using the TRIzol reagent (Invitrogen, Carlsbad, CA, USA) according to the manufacturer’s instructions. Subsequently, 1% agarose gels were utilized to monitor RNA degradation and contamination levels. The purity, concentration, and integrity of the isolated RNA were assessed using the NanoPhotometer spectrophotometer (IMPLEN, Westlake Village, CA, USA), Qubit 2.0 Fluorimeter (Life Technologies, Carlsbad, CA, USA) the and Bioanalyzer 2100 system (Agilent Technologies, Santa Clara, CA, USA). Only high-quality RNA samples were utilized for transcriptome library construction.

### 2.3. Transcriptome Library Construction and Sequencing

The RNA libraries of the mink were generated using the NEBNext Ultra RNA Library Prep Kit for Illumina following the manufacturer’s recommendations. Briefly, The poly-A mRNA was isolated using magnetic beads with attached Oligo (dT). First strand cDNA was synthesized using a random hexamer primer and M-MuLV Reverse Transcriptase (RNase H-). Subsequently, second strand cDNA synthesis was performed using DNA Polymerase I and RNase H. To select cDNA fragments of 370~420 bp in length, the library fragments were purified with the AMPure XP system. The index-coded samples were clustered on a cBot Cluster Generation System using TruSeq PE Cluster Kit v3-cBot-HS (Illumia) according to the manufacturer’s instructions. The sequencing of the libraries was performed using an Illumina HISeq platform (Illumina, San Diego, CA, USA), and 150 bp paried-end reads were generated.

### 2.4. Reads Mapping to the Reference Genome

The raw data in fastq format was initially processed using the fastp software v0.23.2, and the clean data were obtained by filtering the reads with adapter, reads containing poly-N, and low-quality reads from raw data. The calculations of Q20, Q30, and GC content were performed simultaneously on the clean data. All subsequent analyses were conducted exclusively using the high-quality clean data. The paired-end clean reads were aligned to the *Neovison vison* reference genome using the HISAT2 software (version: 2.0.5). The mapped reads of each sample were assembled by StringTie in a reference-based approach for the prediction of novel genes.

### 2.5. Gene Expression Analysis

In addition, the number of reads mapped to each gene was calculated using the HTSeq software v2.0.5, where the fragments per kilo base million (FPKM) of each gene were measured based on the length of the gene and read count that was mapped to the gene. The DESeq software (version: 1.20.0) was used to screen the differentially expressed genes (DEGs) using the read count data. An adjusted *p* value (*q* value) was calculated using Benjamini and Hochberg’s approach for controlling the false discovery rate, where genes with |log2(fold change)| ≥ 2 and *q* < 0.05 were considered as DEGs. The enrichment analysis of GO and KEGG pathways was performed with the clusterProfiler package (version: 3.8.1) in the R software. The local version of the Gene Set Enrichment Analysis (GSEA) analysis tool (version: 3.0) was used to perform GSEA analysis on the GO and KEGG datasets, respectively.

### 2.6. Quantitative RT-PCR Analysis 

The SYBR^®^ Premix Ex Taq™ kit (TaKaRa, Osaka, Japan) was utilized for the performance of a Quantitative RT-PCR (qRT-PCR) assay on a Roche LightCycler480 instrument. The GAPDH gene served as an internal control. Primer sequences used in the experiment are provided in Table 1. The relative mRNA expression level was determined using the 2^−ΔΔCT^ method, and the figure was generated using OriginPro 2018.

## 3. Results

### 3.1. Overview of the Mink Transcriptome

To systematically identify the expressed mRNA and their spatiotemporal expression profiles during muscle growth and development in mink, cDNA libraries were constructed from breast muscle samples of nine silver-blue mink (Y45_1, Y45_2, Y45_3, Y90_1, Y90_2, Y90_3, Y120_1, Y120_2, Y120_3). A total of 447,484,956 raw reads were generated from nine cDNA libraries. After filtering, approximately 65.66 Gb of high-quality clean bases were obtained (Table 2). Of these, 88.81% of the clean reads were mapped to the *Neovison vison* reference genome, with 85.76% that were uniquely mapped. Ultimately, a total of 25,954 genes were identified, including 1404 novel genes. 

**Figure 1:**
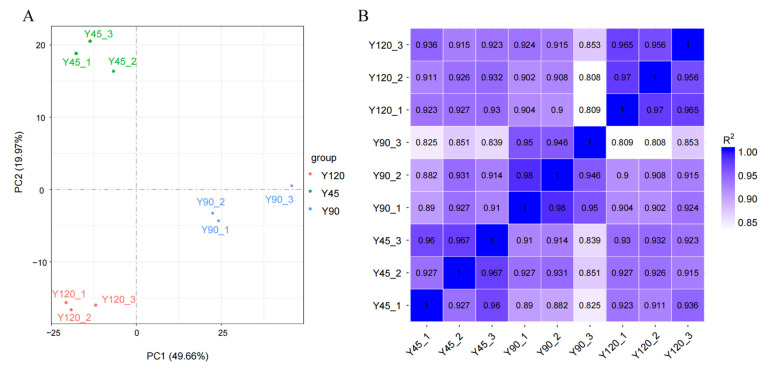
The principal component analysis (**A**) and correlation analysis (**B**) for mink muscle.

The PCA analysis revealed a clear separation among the nine samples, indicating well-defined clusters corresponding to three different time periods (Figure 1A). The squares of Pearson’s correlation coefficient (R^2^) were all greater than 0.92, implying that the samples had a good biological repetition (Figure 1B).

### 3.2. Gene Functional Annotation

**Figure 2:**
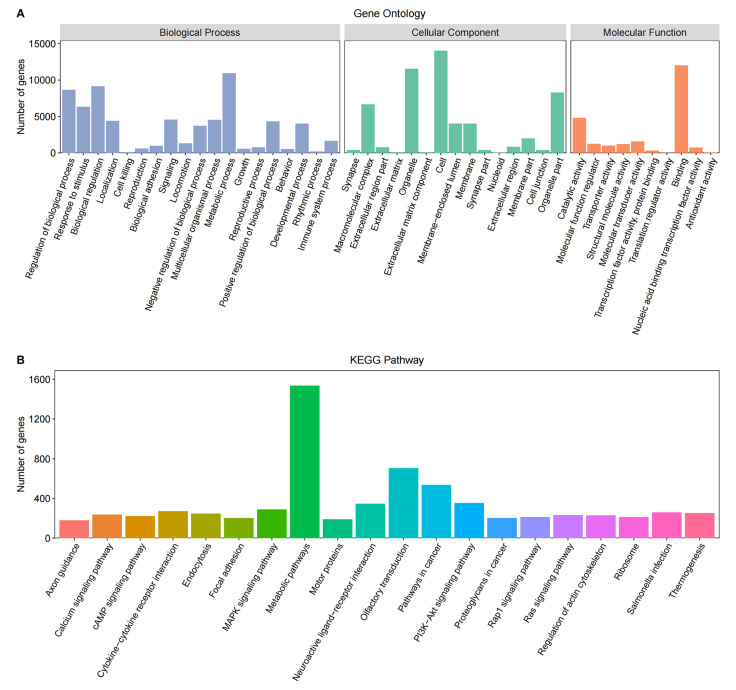
Gene functional annotation based on the Gene Ontology (GO) (**A**) and Kyoto Encyclopedia of Genes and Genomes (KEGG) (**B**) pathway databases.

The gene functional annotation was performed on 25,954 genes, of which 19,992 were successfully annotated into the Gene Ontology (GO) database (Figure 2A). The GO terms primarily encompassed biological processes, such as metabolic processes, biological regulation, and response to stimulus; cellular components, including cell, organelle, organelle part, and macromolecular complex; and molecular functions, such as binding, catalytic activity, and molecular transducer activity.

Among the identified genes, 8120 were successfully annotated into the KEGG Pathway database (Figure 2B), mainly including metabolic pathways, olfactory transmission, pathways in cancer, PI3K-Akt signaling pathway, neuroactive ligand-receptor interaction, MAPK signaling pathway, and Cytokine-cytokine receptor interaction.

### 3.3. Identifying the Differentially Expressed Genes

**Figure 3:**
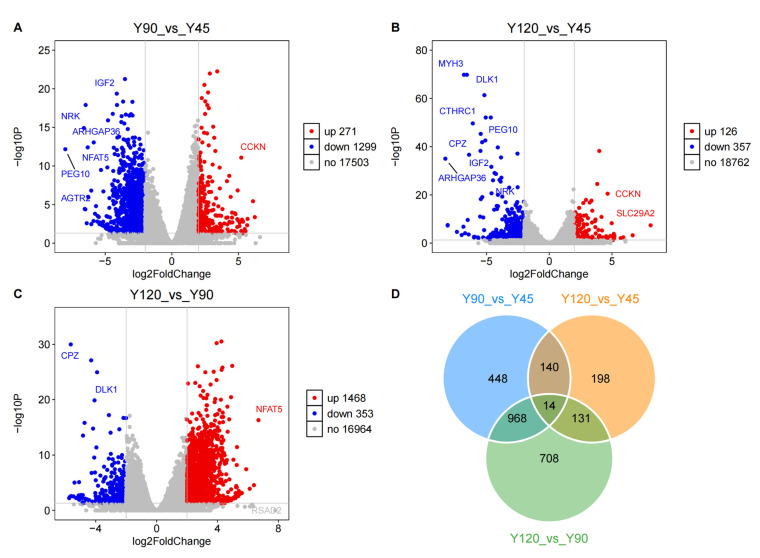
The differential expression genes analysis. (**A**–**C**) Gene expression volcano plots for Y90_vs_Y45, Y120_vs_Y45, and Y120_vs_Y90, respectively; (**D**) The Venn plot of DEGs.

Through pairwise comparisons of muscle samples from three developmental stages, a total of 2607 genes were identified in terms of |log2(fold change)| ≥ 2 and *p*_adj < 0.05. Specifically, there were 1570 DEGs between Y90 and Y45, 483 DEGs between Y120 and Y45, and 1821 DEGs between Y120 and Y90 (Figure 3A–C, Table S1). The volcano plot revealed a significant up-regulation of *PEG10*, *ARHGAP36*, *IGF2*, *NRK*, and *MYH3* with higher fold changes observed at 45 days of age, whereas *PRVA*, *RIT2*, and *SLC29A2* exhibited decreased expression levels at the same time point. The expression level of *NFAT5* at 90 days of age was observed to be lower compared to that at 45 and 120 days of age, indicating a significant temporal variation in its expression; in contrast, *MUSTN1*, a regulator of bone growth and development, showed the inverse pattern. Subsequently, a Venn diagram analysis was conducted on the DEGs. The intersection of DEGs yielded 14 key genes, including *DLK1*, *FBN2*, *MUSTN1*, *NRK*, *PEG10*, *SLC29A2*, *TFRC*, *TCAL7*, *ENSNVIG00000007639*, *ENSNVIG00000009406*, *ENSNVIG00000009488*, *ENSNVIG00000009493*, *ENSNVIG00000020113*, *novel.942* (Figure 3D).

### 3.4. Functional Analysis of the Differentially Expressed Genes

**Figure 4:**
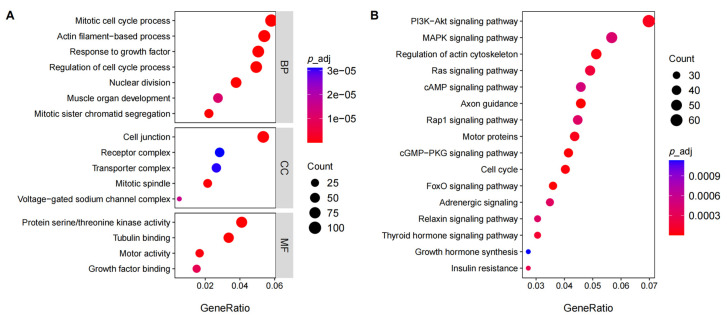
Functional analysis of DEGs. (**A**): the enrichment GO terms in the three categories (Biological Process, Molecular Function, Cellular Component); (**B**): The KEGG pathways with significant enrichment.

The function of DEGs was explored using GO enrichment analysis (Table S2, Figure 4A). For the BP category, the top significance terms were mitotic cell cycle process (*p*_adj = 4.53 × 10^−07^), response to growth factor (*p*_adj = 4.53 × 10^−07^), muscle organ development (*p*_adj = 0.00087). In the case of the CC category, the most abundant GO terms were cell junction (*p*_adj = 6.97 × 10^−05^), mitotic spindle (*p*_adj = 6.97 × 10^−05^), transporter complex (*p*_adj = 0.0012); in the MF category, the DEGs mainly involved protein serine/threonine kinase activity (*p*_adj = 2.63 × 10^−09^), tubulin binding (*p*_adj = 9.26 × 10^−06^), motor activity (*p*_adj = 0.00013), and growth factor binding (*p*_adj = 0.00095). 

The KEGG pathways with significant enrichment are presented in Figure 4B, including axon guidance (*p*_adj = 3.21 × 10^−06^), FoxO signaling pathway (*p*_adj = 8.12 × 10^−06^), cGMP-PKG signaling pathway (*p*_adj = 1.13 × 10^−05^), Cell cycle (*p*_adj = 2.40 × 10^−05^), regulation of actin cytoskeleton (*p*_adj = 3.59 × 10^−05^), motor proteins (*p*_adj = 6.95 × 10^−05^), PI3K-Akt signaling pathway (*p*_adj = 7.36 × 10^−05^), and insulin resistance (*p*_adj = 0.00028) (Table S3).

**Figure 5:**
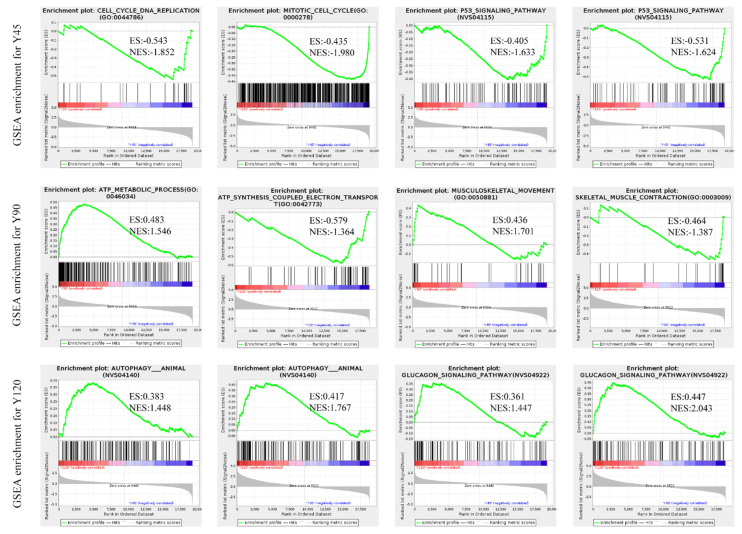
The GSEA analysis result.

In addition to the GO and KEGG enrichment analysis of DEGs, we also performed GSEA-GO and GSEA-KEGG enrichment analysis on all quantitative genes. This comprehensive analysis enabled us to identify the most significantly enriched gene sets in the dataset, providing valuable insights into the cellular processes and pathways most actively involved in the observed cellular functions. The results revealed that the mitotic cell cycle and p53 signaling pathway exhibited enrichment at 45 days of age, while musculoskeletal movement and the ATP metabolic process showed enrichment at 90 days of age. Additionally, the glucagon signaling pathway and autophagy were found to be enriched at 120 days of age (Figure 5, Tables S4 and S5).

### 3.5. qRT-PCR Validation of DEGs

**Figure 6:**
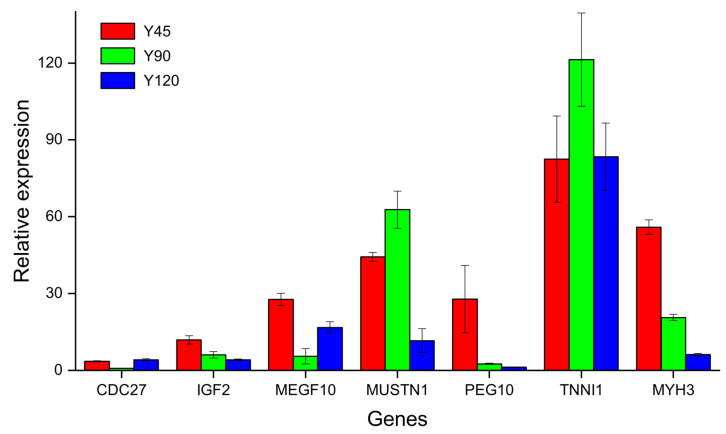
The relative expression levels of seven genes by qRT-PCR.

To further validate the results of RNA-seq, seven DEGs, including cell division cycle 27 (CDC27), insulin like growth factor 2 (IGF2), multiple EGF like domains 10 (MEGF10), musculoskeletal (MUSTN1), paternally expressed 10 (PEG10), troponin I1, slow skeletal type (TNNI1), and myosin heavy chain 3 (MYH3) were selected to perform qRT-PCR. *MUSTN1* and *TNNI1* had higher FPKM in Y90, while *IGF2*, *MEGF10*, *PEG10*, and *MYH3* had higher FPKM in Y45. The relative expression levels of the genes obtained by qRT-PCR, as depicted in Figure 6, exhibited a high degree of concordance with the FPKM values derived from Illumina RNA-seq analysis, thereby confirming the robustness and reliability of the sequencing data.

## 4. Discussion

This study conducted RNA extraction from breast muscle samples collected from silver-blue mink at 45, 90, and 120 days of age during their rapid growth phase. Subsequently, a comprehensive transcriptome database was established to elucidate the genetic regulation of mink’s growth and development.

The expression of 2607 genes showed significant differential regulation, with *PEG10*, *ARHGAP36*, *IGF2*, *NRK*, and *MYH3* exhibiting high foldchange and up-regulation at 45 days of age. Previous studies have demonstrated the crucial roles of *PEG10*, *IGF2*, and *NRK* in embryonic development regulation, characterized by elevated expression levels during the embryonic stage compared to adult individuals [16,17,18,19,20]. This finding enhances our understanding of the up-regulation mechanism of these genes during early mink development. The pivotal role of *IGF2* as a regulator of myogenesis has been widely acknowledged, with its depletion impeding this process [21]. Furthermore, in vitro experiments have demonstrated that a deficiency of *IGF2* within primary skeletal muscle cell-derived myotubes leads to impaired mitochondrial function. However, at 90 days of age, there was a significant increase in the expression levels of *MUSTN1* and *PRVA*, which are crucial genes involved in bone growth and development [22,23]. Therefore, *PEG10*, *IGF2*, and *NRK* are more likely to exert regulatory control over myogenesis during the early postnatal period of mink, while *MUSTN1* is responsible for regulating the proliferation and differentiation of skeletal muscle satellite cells (SMSCs), thereby facilitating skeletal muscle growth. In addition, *DLK1* and *FBN2* were included in a list of 14 shared differentially expressed genes. Furthermore, *DLK1* is an imprinted paternal gene involved in regulating cell growth through encoding a transmembrane protein with multiple epidermal growth factor repeats [24]. It plays a crucial role as a key regulator of mammalian growth and development. Fibrillin-2 (*FBN2*) is a component of connective tissue microfibrils and may be involved in elastic fiber assembly. Its mutations can lead to genetic diseases such as myopathy [25]. The *FBN2* gene has been reported to be associated with height percentile in children and is also a key determinant for skeletal muscle development in Kazakh sheep.

The determination of skeletal muscle fiber numbers primarily occurs during embryonic development, while postnatal changes mainly result from the fusion of muscle satellite cells with fibers, leading to hypertrophy [26]. In the study, 51 DEGs associated with muscle development were identified during the growth and development stages of mink. Notably, *IGF2*, *MYOG*, *MEGF10*, *MYMX*, *MYMK*, *SOX8*, and *PITX1* exhibited upregulated expression at 45 days, with stronger expression in skeletal muscle satellite cells where they play crucial roles in muscle regeneration [7,27,28]. Furthermore, it has been observed that certain genes exhibit a requirement for cooperative interactions in order to ensure their proper functionality. *MYOG* exhibits a similar expression pattern with *MEGF10* and positively regulates *MEGF10* transcription during muscle regeneration; Knockout experiments underscored the indispensable collaboration between *MYMX* and *MYMK* in muscle fiber formation during both embryonic development and adulthood [29,30]. Conversely, *TNNI1*, *TNNI2*, *MYL3*, and *MUSTN1* were up-regulated at day 90, a stage characterized by higher growth rates. It has been demonstrated that *MUSTN1* exhibits the highest expression level during the phase of duck muscle development associated with the maximum relative growth rate [31]. *MYL3*, *TNNI1*, and *TNNI2* have been identified as key regulators of muscle contraction [32,33]. Consequently, these genes are expected to exhibit a positive correlation with the development of breast muscle in mink.

In muscle growth and development, the body precisely regulates the number of cells by regulating key processes such as cell cycle and apoptosis [26]. Cell cycle progression is driven by the dimeric complexes of Cyclin and Cyclin-dependent kinases (CDKs). It is found that at 90 days of age, the genes *ATRX*, *BUB1*, *BUB1B*, *CDK6*, *CDC6*, *CDC27*, *CCNA2*, *E2F2*, *MCM4*, *KNL1*, *RAD21*, and *PPP2R5E* that positively regulate the cell cycle were significantly down-regulated, while *CDKN2C* was up-regulated at this age [34]. It is known that *CDKN2C* is involved in the inhibition of the cell cycle during cell proliferation. Furthermore, we conducted GSEA analysis [35], which primarily focuses on the overall expression pattern of gene sets rather than being limited to DEGs. It theoretically facilitates the identification of genes that may not exhibit significant differential expression but possess crucial biological significance. The results showed that the p53 signaling pathway was significantly enriched in the tissues of 45-day-old minks. In addition to genes involved in cell cycle regulation, genes involved in apoptosis (*BCL2*, *TP53*, *TP73*, *CDKN1A*, *PERP*, *STEAP3*, *PTEN*, *ZMAT3*, and *MDM2*) were significantly associated with this pathway. Although there was no significant difference in the expression of these genes, they may still play an important role in the development of the pectoral muscle tissues of minks.

Many growth factors and cytokines can affect the proliferation and differentiation of satellite cells. It is widely acknowledged that insulin plays an important role in skeletal muscle growth by regulating muscle hypertrophy, protein accumulation, and cell activity. *INSR* is a tyrosine kinase-like insulin receptor that acts as a molecular switch in insulin signaling [36], and the knockdown of *INSR* induces G1/G0 cell cycle arrest and inhibits cell proliferation [37]. *IGF2*, *IGF2R*, *IGF2BP2*, *IGF2BP3*, and *IGFBP6* have also demonstrated significant influence on animal growth and muscle development [38]. Takashi Saito observed a sharp decline in the expression levels of *IGF1*, *IGF2*, *IGFR1*, *IGFR2*, *IGFBP3*, and *IGFBP5* mRNA in masseter muscle between 14 and 19 days postpartum [39]. A transcriptomic analysis of chicken leg muscle showed that the expression levels of *IGFBP3*, *IGFBP5*, and *IGFBP7* decreased at 16 weeks of age [40]; The aforementioned trend is also evident in the findings of our experiments. *IGFBPs*, a family of six or more related proteins that bind IGF with high affinity, could sequester *IGF* to decrease protein synthesis and inhibit muscle cell differentiation. The affinity of *IGFBP6* towards *IGF2* surpasses that of other *IGFBPs*, and the expression level of *IGFBP6* mRNA is highest in muscle tissue [41]. Additionally, it could enhance the muscle differentiation process by triggering predominantly the MAPK pathway in the absence of *IGF2* [42]. Therefore, it is speculated that IGFBP6 regulates the growth of breast muscle independently of *IGF2* during the growth and development of mink. The DEGs also included members of the FGF family, such as *FGF2*, *FGF6*, and *FGFR2*. Notably, *FGF6* is a developmental regulatory gene with highly restricted expression in adulthood that promotes satellite cell proliferation by inducing their entry into the cell cycle [43,44]. The *FGFR2* protein belongs to the fibroblast growth factor receptor family. Its extracellular domain interacts with fibroblast growth factors, initiating a cascade of downstream signals that ultimately regulate mitogenesis and differentiation. Hence, similar to *IGF2*, *FGFs* also tend to play a more important regulatory role in the early growth and development of mink.

## 5. Conclusions

This study utilized RNA-seq technology and bioinformatics methods to characterize the gene expression profile of silver-blue mink muscle tissue. The results indicate that a total of 25,954 gene expression profiles were obtained during the growth and development stages of mink at 45, 90, and 120 days, with 2607 genes showing significant differential expression. Most differentially expressed genes (DEGs) were associated with the mitotic cell cycle, response to growth factors, muscle organ development, and insulin resistance. Subsequent analysis revealed the upregulation of genes related to embryonic development, cell cycle regulation, and the FGF family at 45 days in mink. Conversely, genes involved in skeletal muscle development and the negative regulation of the cell cycle were found to be upregulated at 90 days. These findings provide valuable insights into the regulatory mechanisms underlying mink growth and development.

## Figures and Tables

**Table 1:** Primers for quantitative real-time PCR.

Gene	Gene Description	Primers Sequence (from 5′ to 3′)
*CDC27*	Cell division cycle 27	F: TCTCCACAATCACACCTCAGATCC R: TTCACGAAGAAGGCTCATCAAACC
*IGF2*	Insulin like growth factor 2	F: GCCCTTCTGGAGACCTACTGTG R: AGGTGTCGTATTGGAAGAACTTGC
*MEGF10*	Multiple EGF like domains 10	F: TTCCGAGGCACCACTTGTCAG R: CCAGGCAGGCAGTCACAGAG
*MUSTN1*	Musculoskeletal	F: GCCAAGAACCAGGAGATCAAGTC R: TCGGCTGCCACTGAACACC
*PEG10*	Paternally expressed 10	F: GATGGACATGGACGATCACTCTATG R: TGCGGCGGCGGATACTG
*TNNI1*	Troponin I1, slow skeletal type	F: GTGGAGGTGGTGGATGAGGAG R: CCCGACGCAGTGGTGGAC
*MYH3*	Myosin heavy chain 3	F: CGTCCTGGATGATCTACACCTACTC R: TTCTTGCCTCGGTAGCCTTCC

**Table 2:** Summary of the sequencing data of the nine silver-blue mink.

Samples	Raw Reads	Clean Reads	Clean Bases (Gb)	Total Mapped	Uniquely Mapped
Y45_1	46,417,512	45,271,560	6.79	87.79%	84.94%
Y45_2	55,331,762	54,289,360	8.14	90.09%	87.11%
Y45_3	50,207,396	49,355,610	7.4	89.09%	86.20%
Y90_1	50,046,636	49,083,374	7.36	88.26%	84.96%
Y90_2	48,711,358	47,736,736	7.16	88.75%	85.48%
Y90_3	50,268,714	48,472,740	7.27	87.95%	85.02%
Y120_1	48,166,278	47,430,618	7.11	90.19%	87.36%
Y120_2	48,847,066	47,665,894	7.15	87.26%	83.75%
Y120_3	49,488,234	48,537,954	7.28	89.67%	86.8%

## Data Availability

Data are contained within the article.

## Supplementary Materials

The following supporting information can be downloaded at: https://www.mdpi.com/article/10.3390/biology13050283/s1, Table S1: List of the identified differentially expressed genes (DEGs); Table S2: GO functional enrichment analysis of differentially expressed genes (DEGs); Table S3: KEGG functional enrichment analysis of differentially expressed genes (DEGs); Table S4: The P53 signaling pathway in Y90_vs_Y45 for GSEA-KEGG analysis; Table S5: The P53 signaling pathway in Y120_vs_Y45 for GSEA-KEGG analysis.
